# Inflammatory microbes and genes as potential biomarkers of Parkinson’s disease

**DOI:** 10.1038/s41522-022-00367-z

**Published:** 2022-12-24

**Authors:** Shiqing Nie, Jichen Wang, Ye Deng, Zheng Ye, Yuan Ge

**Affiliations:** 1 State Key Laboratory of Urban and Regional Ecology, Research Center for Eco-Environmental Sciences, Chinese Academy of Sciences, Beijing, 100085 China; 2 University of Chinese Academy of Sciences, Beijing, 100049 China; 3 Key Laboratory of Environmental Biotechnology, Research Center for Eco-Environmental Science, Chinese Academy of Sciences, Beijing, 100085 China; 4 Institute of Neuroscience, Center for Excellence in Brain Science and Intelligence Technology, Chinese Academy of Sciences, Shanghai, 200031 China

**Keywords:** Microbiota, Clinical microbiology

## Abstract

As the second-largest neurodegenerative disease in the world, Parkinson’s disease (PD) has brought a severe economic and medical burden to our society. Growing evidence in recent years suggests that the gut microbiome may influence PD, but the exact pathogenesis of PD remains unclear. In addition, the current diagnosis of PD could be inaccurate and expensive. In this study, the largest meta-analysis currently of the gut microbiome in PD was analyzed, including 2269 samples by 16S rRNA gene and 236 samples by shotgun metagenomics, aiming to reveal the connection between PD and gut microbiome and establish a model to predict PD. The results showed that the relative abundances of potential pro-inflammatory bacteria, genes and pathways were significantly increased in PD, while potential anti-inflammatory bacteria, genes and pathways were significantly decreased. These changes may lead to a decrease in potential anti-inflammatory substances (short-chain fatty acids) and an increase in potential pro-inflammatory substances (lipopolysaccharides, hydrogen sulfide and glutamate). Notably, the results of 16S rRNA gene and shotgun metagenomic analysis have consistently identified five decreased genera (*Roseburia*, *Faecalibacterium*, *Blautia*, *Lachnospira,* and *Prevotella*) and five increased genera (*Streptococcus*, *Bifidobacterium*, *Lactobacillus*, *Akkermansia,* and *Desulfovibrio*) in PD. Furthermore, random forest models performed well for PD prediction based on 11 genera (accuracy > 80%) or 6 genes (accuracy > 90%) related to inflammation. Finally, a possible mechanism was presented to explain the pathogenesis of inflammation leading to PD. Our results provided further insights into the prediction and treatment of PD based on inflammation.

## Introduction

Parkinson’s disease (PD) is an incurable, progressive, and chronic neurodegenerative disease characterized by the formation of Lewy bodies (mainly formed by misfolded α-synuclein) and the loss of dopaminergic neurons in the substantia nigra^1^. More than 6 million individuals worldwide were diagnosed with PD. PD alters dopaminergic, noradrenergic and serotonergic neurons in the brain, causing a drop in dopamine levels and premotor and non-motor symptoms, including akinesia, rigidity, balance difficulties, tremor, as well as neuropsychiatric, cognitive, autonomic and sensory disturbances. These non-motor symptoms can appear years or even decades before motor symptoms appear, but are often unrecognized^2^, resulting in PD patients not receiving timely treatment. Although PD has brought great medical and social burdens, its specific pathogenesis is still unclear. Numerous measurements have been used to diagnose PD and mainly include positron emission tomography, cerebrospinal fluid tests, and clinical symptoms^3^. However, positron emission tomography is quite costly and the reproducibility and reliability of the cerebrospinal fluid tests have been suspected. Therefore, it is imperative to further explore the pathogenesis of PD and find reliable and cheap biomarkers.

In recent years, it has been proposed that the human gastrointestinal microbiota is one of the most important pathogenic mechanisms of many neurodegenerative diseases^4,5^. Gut microbiota encodes millions of genes and produces thousands of metabolites, affecting the metabolism of the host^4,6,7^. Substantial evidence suggests a bidirectional interaction between the gastrointestinal microbiota and the central nervous system, known as the “gut–microbiota–brain axis”^8^. Multiple “gut–microbiota–brain axis” pathways exist, including molecules with neuroendocrine activity produced by microbes (such as gamma-aminobutyric acid and serotonin) and the gut microbial community influenced by the central nervous system^8^. These connections form a feedback loop between human physiology and the state of the microbial community. In recent years, the gut microbiota has been proven to play a vital role in the progression of PD in animal models through the gut–microbiota–brain axis^9,10^.

Multiple studies have described prodromal symptoms (gastrointestinal motility disorders) affecting the quality of life of patients with PD, including delayed gastric emptying and chronic constipation^11^. Intestinal symptoms (e.g., constipation) often precede motor symptoms, indicating a possible pathogen in the gut of PD patients. Based on neuropathology, Braak et al.^12^ suggested that PD may be caused by an enteric pathogen that can cross the intestinal mucosal barrier and enteric neurons, and ultimately enter the central nervous system via the vagus nerve. The hallmarks of PD, Lewy bodies and misfolded α-synuclein proteins, were found in both the central and enteric nervous systems^13^. The transport of α-synuclein in the gut–microbiota–brain axis and the newly discovered vagal pathway may induce or accelerate the progression of PD^11^. Removing the vagus nerve appears to reduce the risk of PD^14,15^. In a landmark study, Sampson et al. demonstrated that the microbiome itself can trigger or delay the motor symptoms of PD in mice^9^. Microbiota may facilitate α-synuclein diffusion, since the gut microbiota can secrete extracellular amyloid, and proteins such as PrPSc, Tau, and α-synuclein can spread in the body like prions^16^. Aggregation-prone proteins such as α-synuclein and Tau spread throughout the body during microbial colonization, biofilm formation and infection^17^. In PD patients, several indicators of symptom severity were positively correlated with microbial alpha and beta diversity indices^18^. The above evidence suggests that dysbiosis of gastrointestinal microbiota may provide an interesting clue to explore the pathogenesis of PD and become a new diagnostic and therapeutic target.

We hypothesized that PD patients in different countries and regions share common microbial and metabolic characteristics. In this study, the bacterial communities and metabolic pathways of PD were characterized by collecting massive open-access 16S rRNA gene and shotgun metagenomic data from extensive studies. Our goal is to understand the underlying microbial community and metabolic patterns in the gut of PD patients, then reveal the pathogenesis of PD and construct a model to predict PD.

## Results

### Changes in bacterial communities

**Fig. 1:**
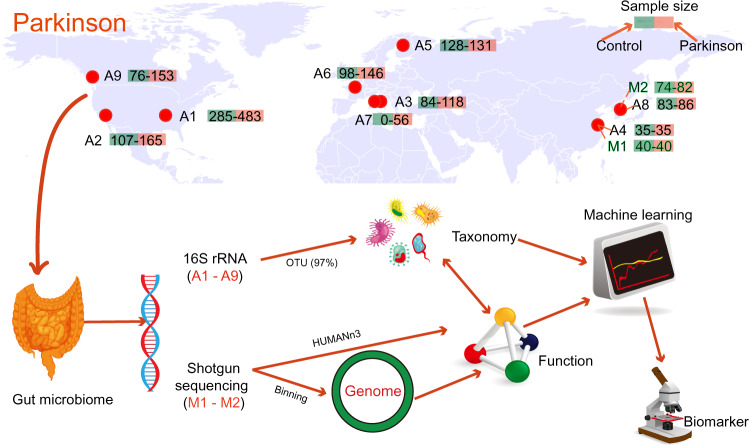
Study design. Nine datasets (A1–9) including 2269 16S rRNA gene samples and two datasets (M1-M2) including 236 shotgun metagenomic samples were collected from 11 studies. Taxonomy was inferred from 16s rRNA gene based on the operational taxonomic unit (OTU), the function was inferred from shotgun sequencing, and the genome was obtained by binning (metagenome-assembled genomes). Finally, the biomarker was found using machine learning.

After quality control, nine datasets (A1–9) including 2269 16S rRNA gene amplicon samples (1373 PD and 896 healthy controls) and two datasets (M1-2) including 236 shotgun sequencing metagenomic samples (122 PD and 114 healthy controls) were collected by searching the keywords “Parkinson” and “microbes” in the National Center for Biotechnology Information (NCBI) SRA database and Google Scholar (Fig. 1 and Supplementary Table 1). The resulting merged operational taxonomic unit (OTU) table contained 3847 taxa from 2269 16S rRNA gene samples (Supplementary Table 2). To explore whether the composition of the gut bacteria differed between the PD patients and healthy control, firstly, three α-diversity indexes (Shannon, Simpson, Pielou) were calculated, and Principal coordinates analysis (PCoA) was performed. The results (Supplementary Fig. 1) showed that there was no significant difference in α-diversity (0.89 > *p* > 0.62, Wilcoxon rank-sum test) and β-diversity (*p* = 0.72, analysis of similarities) between PD and Healthy control.

**Fig. 2:**
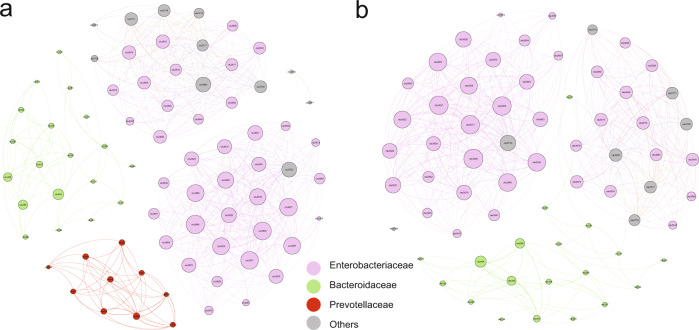
Co-occurrence networks. Co-occurrence networks were calculated based on Spearman correlations at the operational taxonomic unit (OTU) level. The more connections, the larger the node (degree). Only strong (*r* > 0.7) and significant (*p* < 0.05) correlations are shown in the figure and nodes with few connections (<5) were removed. **a** Healthy controls. **b** Patients with Parkinson’s disease (PD). Nodes were colored by family.

To explore the potential taxon co-occurrence pattern in PD, Spearman’s correlations between the microbial taxa (OTU) were calculated and visualized based on the combined dataset (A1–9). There was an obvious difference in the network structure between PD and healthy control (Fig. 2 and Supplementary Fig. 2). The results revealed a less number of nodes and links in the PD network (Supplementary Table 3). After removing nodes with few connections (<5), the network of healthy control contained four main modules while PD had only three (Fig. 2). The nodes in the network were dominated by *Enterobacteriaceae*, *Bacteroidaceae* and *Prevotellaceae*. It is worth noting that *Prevotellaceae* (*Prevotella*) did not appear in the PD network (Fig. 2).

**Fig. 3:**
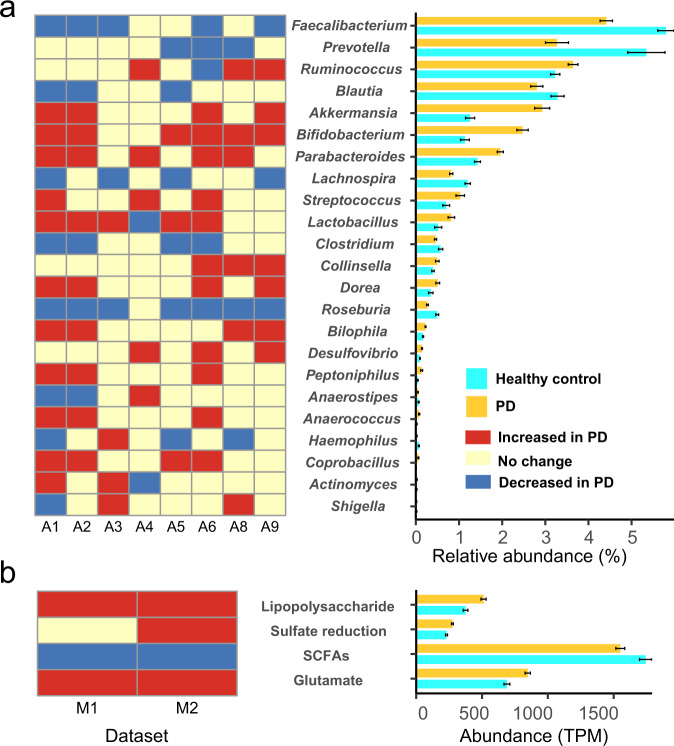
Differences in microorganisms and metabolic pathways between healthy controls and patients with Parkinson’s disease (PD). **a** Genera with significant differences in relative abundance. Amplicon datasets (A1–9). **b** Metabolic pathways (related to inflammation) with significant differences in transcripts per million (TPM) abundance. Metagenomic datasets (M1–2). SCFAs: short-chain fatty acids. The error bar represents the standard error of the mean abundance in each bar plot.

At the genus level, the relative abundances of 23 genera (Fig. 3a) were significantly different between healthy control and PD in at least three datasets (*p* < 0.05, Wilcoxon rank-sum test). Amplicon dataset 7 (A7) can not be analyzed separately for difference statistics because it only contains PD samples. These 23 genera also share the same variance in the combined dataset (A1–9) except *Collinsella*, *Ruminococcus*, *Dorea*, *Shigella,* and *Anaerostipes* (Supplementary Fig. 3). Five genera (*Roseburia*, *Faecalibacterium*, *Blautia*, *Lachnospira,* and *Prevotella*) are well-known producers of short-chain fatty acids (SCFAs) in the gut^19,20^, and their abundances were significantly reduced in PD. These five genera may be associated with anti-inflammation in PD. *Streptococcus*^21^ is an opportunistic pathogen, and its relative abundance was significantly increased in PD. Three genera (*Bifidobacterium*^22^, *Lactobacillus*^23^, and *Akkermansia*^24^) are probiotics, but their abundances were significantly increased in PD patients. *Desulfovibrio*^25^ predominates among intestinal sulfate-reducing bacteria with the ability to produce hydrogen sulfide (H_2_S), and its abundance was significantly increased in PD. These five genera may be associated with pro-inflammation in PD. It may seem ironic that these probiotics were elevated in PD patients, but they may also act as opportunistic pathogens and even cause damage in immunocompromised individuals under certain conditions^22–24^. SCFAs are anti-inflammatory under certain conditions, while H_2_S promotes intestinal inflammation. Except for these ten genera, there seemingly are no reports related to PD in other genera. The results above suggested that inflammation may play a key role in the pathogenesis of PD. Previous studies have similarly shown a strong link between PD and inflammation-associated bacteria^26,27^. Therefore, metabolic pathways related to inflammatory metabolism and the ten potential inflammation-related genera mentioned above were further analyzed.

### Significant changes in inflammatory metabolic pathways

Functional annotation was performed using HUMANn3 based on the metagenomic combined dataset (M1–2), and then potential inflammatory metabolism-related pathways and genes were selected for further analysis. By reviewing the literature on Google Scholar with “Parkinson” and “inflammation” as keywords, four commonly reported metabolic pathways (SCFAs, sulfate reduction, lipopolysaccharide, and glutamate) related to intestinal inflammation were identified^18,19^. SCFAs resist intestinal inflammation, while sulfate reduction, lipopolysaccharide, and glutamate promote intestinal inflammation. The genes of these four metabolic pathways were determined through the MetaCyc database^28^. Finally, 7958 genes (UniRef90) for these four metabolic pathways were extracted from the results of HUMANn3. The transcripts per million (TPM) abundances^29^ of genes belonging to the same metabolic pathway were summed as the abundance of that metabolic pathway.

The TPM abundances of 63 genes were significantly changed (*p* < 0.01, Wilcoxon rank-sum test, Supplementary Fig. 3b) in the combined dataset (M1–2). Among them, 19 SCFAs genes were significantly decreased, and all sulfate reduction (4), lipopolysaccharide (4) metabolism and glutamate metabolism (18) genes were increased in PD. Moreover, the SCFAs pathway was significantly decreased, while the sulfate reduction, lipopolysaccharide and glutamate metabolism pathways were increased in PD (*p* < 0.01, Wilcoxon rank-sum test, Fig. 3b).

**Fig. 4:**
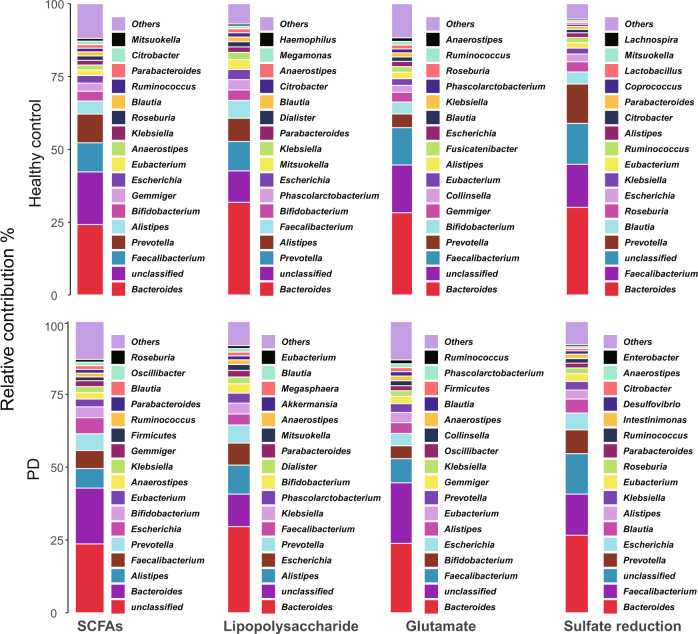
The contribution rate of genera to the four inflammation-related metabolic genes. The value is the mean contribution rate in two Metagenomic datasets (M1–2). Parkinson’s disease (PD). SCFAs short-chain fatty acids.

HUMANn3 provided the correspondence between genes and microorganisms, which allows us to analyze the source of genes^29^. The source of the genes of the four metabolic pathways was shown in Fig. 4 and Supplementary Table 4 at the genus level in the metagenomic combined dataset (M1-2). *Bacteroides*, *Faecalibacterium*, *Prevotella*, and *Alistipes* had the greatest contribution to the four metabolic pathway genes. 128 genera can provide SCFAs metabolism genes, including *Roseburia*, *Faecalibacterium*, *Blautia*, *Lachnospira,* and *Prevotella*. 114 genera can provide glutamate metabolism genes, 89 genera can provide lipopolysaccharide metabolism genes, and 73 genera can provide sulfate reduction genes, including *Streptococcus, Bifidobacterium*, *Lactobacillus*, *Akkermansia,* and *Desulfovibrio*. It is worth noting that *Roseburia* not only provided a large number of SCFAs genes, but its contribution to SCFAs genes decreased (*p* < 0.05, Wilcoxon rank-sum test) in PD, from 1.5% in healthy control to 0.9% in PD, and the contribution of *Desulfovibrio* to sulfate reduction genes was significantly increased (*p* < 0.05, Wilcoxon rank-sum test), from 0.04% in healthy control to 1% in PD (Fig. 4 and Supplementary Table 4).

### Genome reconstruction

**Fig. 5:**
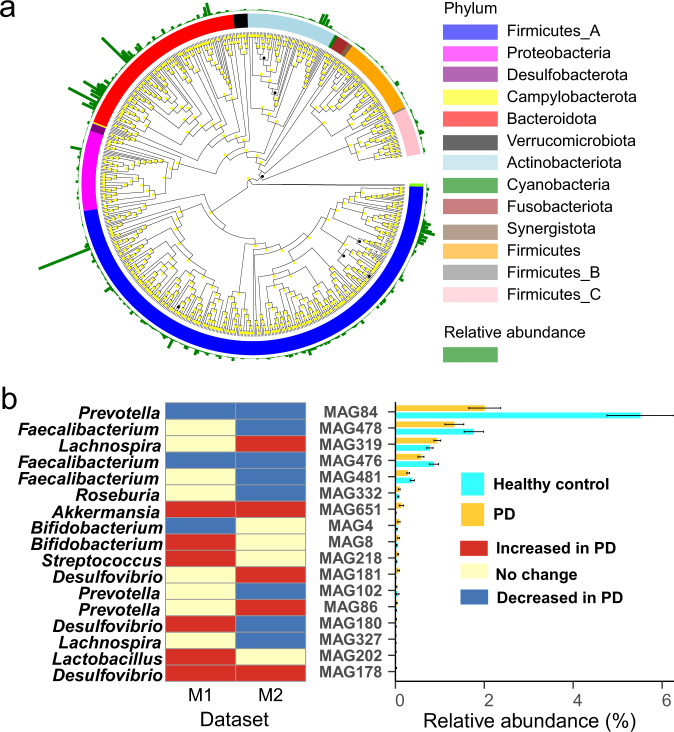
Metagenome-assembled genomes analysis. **a** The maximum-likelihood phylogenetic tree of the 654 metagenome-assembled genomes (MAGs). The tree was based on the 120 bacterial (122 archaeal) concatenated ribosomal proteins, colored by phyla. Bootstrap values were calculated based on 1000 replicates, and the value higher than 80% were marked in yellow. **b** The 17 MAGs with significant differences between Parkinson’s disease (PD) and healthy groups in at least one Metagenomic dataset (M1–2). The error bar represents the standard error of the mean relative abundance in each bar plot.

To further explore the correspondence between genes and microorganisms, binning analysis was performed. Binning yielded 654 metagenome-assembled genomes (MAGs) with high-quality (completeness > 80%, contaminate < 5%, 652 bacterial MAGs and 2 archaeal MAGs) from the combined dataset (M1-2), including 13 phyla (Fig. 5a and Supplementary Table 5). Most MAGs are *Firmicutes_A* (318) and *Bacteroidota* (116), and most *Bacteroidota* MAGs had high relative abundance. For the combined dataset (M1-2), the relative abundances of 242 MAGs were significantly different between PD and healthy groups (*p* < 0.05, Wilcoxon rank-sum test), and the number of pro-inflammatory and anti-inflammatory genes contained in each MAG is shown in Supplementary Fig. 4 and Supplementary Table 5.

As shown in Fig. 5b, 17 MAGs related to inflammation were significantly different between PD and healthy groups (*p* < 0.05, Wilcoxon rank-sum test). The results of MAGs analysis (Fig. 5b) were generally consistent with amplicon analysis (Fig. 3a) that the relative abundances of potential anti-inflammatory MAGs were significantly decreased in PD in at least one dataset (M1 and M2) and potential pro-inflammatory MAGs were significantly increased.

### Predicting PD with inflammatory microbes and genes

**Fig. 6:**
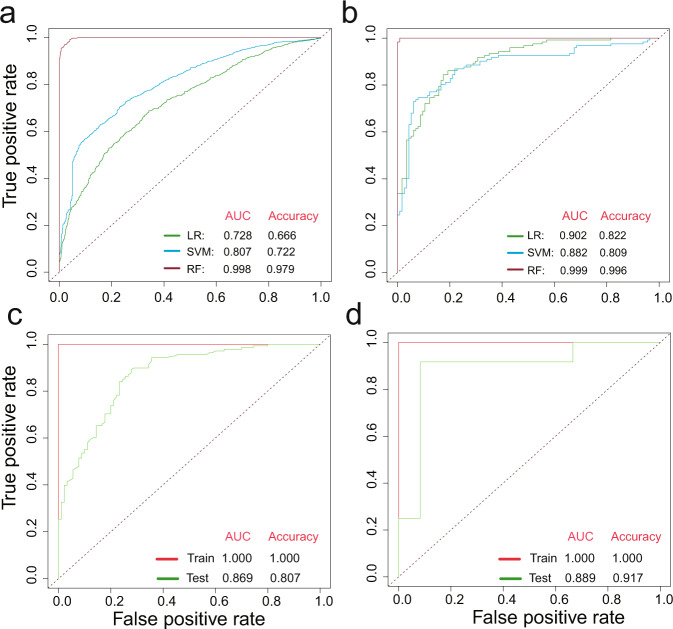
The prediction models constructed by machine learning. The receiver operating characteristic (ROC) curve of models was calculated based on 32 genera (**a**) and 63 genes (**b**) with three different methods (i.e., logistic regression (LR), support vector machines (SVM), and random forests (RF)). The performance of the optimal model using 11 genera (**c**) or 6 genes (**d**) on training and test sets.

The above results have demonstrated that the relative abundances of potential inflammation-related microorganisms and genes in PD changed significantly. These differential microorganisms and genes were then used to build classification models through three machine learning methods (logistic regression (LR), support vector machines (SVM), and random forests (RF)), and the receiver operating characteristic (ROC) curve and the area under the curve (AUC) were used to evaluate the model performance. Based on the 32 genera (Supplementary Fig. 3a) which significantly changed in PD, the classification model with high accuracy can be obtained (Fig. 6a), and the performance of RF (AUC = 0.99, Accuracy = 97%) was better than that of SVM (AUC = 0.80, Accuracy = 72%) and LR (AUC = 0.72, Accuracy = 66%). Based on the 63 genes (Supplementary Fig. 3b) which significantly changed in PD, the classification model with high accuracy also can be obtained (Fig. 6b), and the performance of RF (AUC = 0.99, Accuracy = 99%) was better than that of SVM (AUC = 0.88, Accuracy = 80%) and LR (AUC = 0.90, Accuracy = 82%). Therefore, RF was chosen for further analysis.

Considering that it is necessary to minimize the measured indicators to reduce the cost in the actual diagnosis process, the model was further optimized. Firstly, the MeanDecreaseGini index of the 32 genera and the 63 genes was calculated. The larger the MeanDecreaseGini, the more important it is to the model. Then, the genes were sorted according to MeanDecreaseGini from large to small (Supplementary Fig. 5), and different numbers of genera or genes from the front were selected for modeling. Ultimately, the optimal model was obtained based on 11 genera or 6 genes (Supplementary Fig. 6). The optimized models (Fig. 6c, d) had good performance on both the training set (AUC = 1, Accuracy = 100%, based on 11 genera or 6 genes) and the test set (AUC = 0.869, Accuracy = 80.7%, based on 11 genera; AUC = 0.889, Accuracy = 91.7%, based on 6 genes). The importance of each variable in the optimal model is shown in Supplementary Fig. 7.

## Discussion

This study is the largest meta-analysis of the gut microbiome in PD to date, which provided for the first time an integrative analysis of 16S rRNA gene and shotgun metagenomic data on PD and a detailed exploration of how alterations in gut bacterial composition and function affect PD. Firstly, there was no significant difference in bacterial alpha and beta diversity between PD patients and healthy controls (Supplementary Fig. 1). Since the samples in this study came from various countries and regions, the difference may be masked by some confounding factors such as dietary habits, region, gender, sampling method, etc^23^. However, the co-occurrence networks (Fig. 2 and Supplementary Fig. 2) showed that the co-occurrence network of PD was obviously changed. Of note, *Prevotella* was only present in the network of healthy control after removing the nodes with few connections (<5). In addition, Fig. 3a and Supplementary Fig. 3a showed that the relative abundances of potential anti-inflammatory bacteria were decreased and potential pro-inflammatory bacteria were increased in PD.

Therefore, we hypothesized that inflammation is a factor contributing to PD. Then, the genes of metabolic pathways associated with inflammation were further analyzed. Here, SCFAs metabolism was a potential anti-inflammatory metabolic pathway, and potential pro-inflammatory metabolic pathways include lipopolysaccharide, H_2_S and Glutamate metabolism. Our results showed that (Supplementary Fig. 3b and Fig. 3b) more than half of the potential anti-inflammatory genes and pathways were significantly decreased in PD, while that of all the potential pro-inflammatory genes and pathways were significantly increased. Furthermore, the gene source and binning analysis (Figs. 4 and 5) clarified which microorganisms provided these genes at the genus and genome level. These results further demonstrated that the significantly altered gut microbes mentioned above indeed have potential anti- or pro-inflammatory functions. Finally, the optimal RF models were obtained with high accuracy (>80%) to distinguish PD from healthy control based on the 11 genera or the 6 genes related to inflammation. Interestingly, the model based on the 6 genes outperformed the model based on the 11 genera (Fig. 6). However, the diagnosis of PD remains a problem since many clinical characteristics of PD overlap with other neurodegenerative diseases^30^.

SCFAs^31,32^ are the most common gut microbial metabolites, of which over 95% are composed of butyrate, propionate and acetate. SCFAs have numerous physiological functions such as manipulating the maturation of microglia (immune effector cells) in the central nervous system, strengthening intestinal epithelial cells, and reducing inflammation risk^19^. SCFAs also can bind to G protein-coupled receptors such as GPR41, GPR109A and GPR43, and exert anti-inflammatory effects by activating regulatory T cells^33^. Previous studies also demonstrated that the SCFAs-producing bacteria were reduced in PD^32^. A study in Germany showed that PD patients had reduced SCFAs in their feces^34^. Reduced SCFAs (i.e., butyrate, acetate, and propionate) in feces were also found using both a targeted gas chromatography platform and an untargeted nuclear magnetic resonance metabolomics platform^35^. In this study, five potential SCFAs producers (*Roseburia*, *Faecalibacterium*, *Blautia*, *Lachnospira*, and *Prevotella*) were decreased in PD, which was consistent with previous studies^19,20,32^. However, the change of *Prevotella* in PD was controversial in previous literature. For example, the previous meta-analysis^19,32^ reported that most studies found a decreased *Prevotella* in PD, but opposite results were also obtained in some studies. Wallen et al.^23^ claimed that this contradiction may be due to the use of different taxonomic classifiers in different studies (Supplementary Table 1). To avoid this contradiction, the same taxonomic classifier was used for the nine amplicon datasets in this study.

Lipopolysaccharide is a component of the outer wall of Gram-negative bacteria. Bacterial lipopolysaccharide was proven to alter miRNA expression in macrophages, resulting in a cascade of inflammatory responses^22^. This process will lead to mitochondrial dysfunction, iron accumulation, dopamine depletion, and neuroinflammation that may further drive the development of PD^36^. Lipopolysaccharide can also lead to toll-like receptor (TLR) activation, causing gut and brain inflammation and barrier deficiency in PD. PD mouse models indicated that lipopolysaccharide can result in a loss (34%) of dopamine neurons and a heavy pro-inflammatory response through glial activation and increased TNF-α, IL-10, IL-6, and IL-1^20^. Pietrucci et al.^37^ also reported a high level of lipopolysaccharide synthesis in PD, which is consistent with the findings of this study. However, it should be noted that not all bacteria that produce lipopolysaccharides will promote inflammation. Therefore, in this study, these bacteria which increased in PD and contain lipopolysaccharides synthesis genes were thought to have the potential to promote inflammation in PD. However, it is worth noting that these bacteria can not be considered as “classical intestinal pro-inflammatory bacteria”, because there was no direct evidence to prove that they have a pro-inflammatory effect. Similarly, the other changed bacteria mentioned in this paper can not simply be considered as “classic inflammation-associated bacteria”, such as *Lactobacillus* and *Bifidobacteria*.

As a gas neurotransmitter, H_2_S is produced by sulfate reduction of certain gut microbes (such as *Desulfovibrio*), which can affect neuronal signaling at low concentrations and be severely toxic at high concentrations^25^. High concentrations of H_2_S can help to release mitochondrial cytochrome c into the cytoplasm, where the cytochrome can then form α-synuclein free radicals, ultimately triggering the aggregation of α-synuclein^38^. In addition, H_2_S can increase the level of iron in the cytoplasm, which will further lead to α-synuclein aggregation. High concentrations of H_2_S also can inhibit intestinal motility and cause constipation, serious central nervous system dysfunction and even death^39^. H_2_S can reduce disulfide bonds in the mucosal layer of the enteric epithelium, thereby disrupting the intestinal barrier^40,41^. *Desulfovibrio* is the dominant sulfate reduction bacteria in the human gut^25^, also producing lipopolysaccharide and Fe_3_O_4_. *Desulfovibrio* has the capacity to reduce ferric iron to ferrous iron by the periplasmic [FeFe]-hydrogenase, which is present in almost all *Desulfovibrio*, and thus can produce Fe_3_O_4_^42^. Exposed Fe_3_O_4_ nanoparticles have been proven to stimulate α-synuclein aggregation^43^. Of note, multiple lines of evidence in this study (Figs. 3, 4 and 5) have repeatedly confirmed that *Desulfovibrio* can provide sulfate reduction genes and its relative abundance is significantly increased in PD which was consistent with the previous study^25^.

Glutamate acts as an excitatory neurotransmitter, causing excitatory responses^44^. Glutamate is the richest excitatory neurotransmitter in the human brain, which is 1000 times higher than other important excitatory neurotransmitters such as serotonin, dopamine, and norepinephrine^45^. Excessive glutamate induces overstimulation of glutamate receptors and increases intracellular Na^+^ and Ca^2+^ concentrations, which can directly lead to neuronal damage and cell death. Inflammation is known to induce glutamate excitotoxicity, and a high level of glutamate will cause elevated harmful amino acid metabolite (phenylacetylglutamine) that further exacerbate inflammation^46^. For these reasons, glutamate synthesis was defined as a potential pro-inflammatory pathway in the present study. However, this does not imply that glutamate is a formal inflammatory factor.

The combination of a mucus layer composed of mucins with the gut microbiota is considered as a gut biofilm. The gut biofilm can prevent intestinal damage, thereby preventing intestinal permeability. Decreased *Blautia*, *Roseburia,* and *Faecalibacterium* in this study (Fig. 3 and Supplementary Fig. 3) are commensal bacteria involved in gut biofilm^24^. Increased *Akkermansia* (Figs. 3 and 5) which has been reported^47^ may lead to intestinal permeability, as this genus requires mucus for energy, leading to biofilm disruption. Defects in the gut barrier increase the risk of systemic exposure to inflammatory microbial products such as lipopolysaccharide^18^. In addition, increased lipopolysaccharide and decreased lipopolysaccharide-binding protein were detected in the blood, supporting the existence of a defect in the intestinal barrier^48^.

Growing research supported inflammation as a hallmark of PD^49,50^. Raised numerous inflammatory molecules in the brain and blood were founded in PD patients^1,49^. Excess inflammatory microbial products (such as lipopolysaccharides) may cause damage to the intestinal barrier, further leading to systemic inflammation^51^. Compared with healthy controls, PD patients had higher levels of zonulin and alpha-1-antitrypsin, markers of intestinal permeability. Researchers found that the longer the course of PD, the less anti-inflammatory bacteria and more pathogenic bacteria^20^. High-level intestinal inflammation can activate glial cells and enteric neurons, and lead to α-synuclein misfolding and aggregation^31^.

**Fig. 7:**
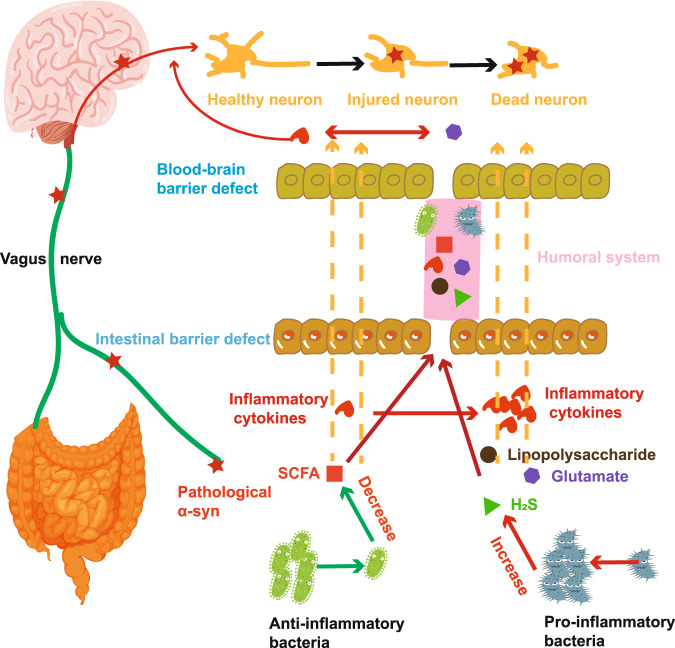
The potential pathogenesis of Parkinson’s disease based on inflammation. Changes in bacterial abundance may lead to decreased anti-inflammatory substances, such as short-chain fatty acids (SCFAs), and increased pro-inflammatory substances (Lipopolysaccharide, Glutamate, H_2_S), resulting in the accumulation of pathological α-synuclein (α-syn), increased inflammatory cytokines, intestinal inflammation and intestinal barrier defects. These substances may reach the brain through the vagus nerve or humoral system, and may eventually cause Parkinson’s disease (PD).

Based on the above analysis, we proposed a potential model to elucidate how inflammation contributes to PD (Fig. 7). In the gut of PD patients, the changed bacteria may lead to a decrease of anti-inflammatory factors (such as SCFAs) and an increase of pro-inflammatory factors (such as lipopolysaccharide, H_2_S and glutamate), causing intestinal inflammation and intestinal barrier damage. Intestinal barrier defect induces leakage of the microbiota and its metabolites (such as lipopolysaccharide, H_2_S and glutamate) into the body, prompting the production of inflammatory cytokines and pathological α-synuclein, further causing blood-brain barrier deficiency. These microorganisms and their metabolites can cross the blood-brain barrier through the humoral system, resulting in microglia and astrocytes activation and brain neuroinflammation^9^. Pathologic α-synuclein may be transmitted to the brain through the vagus nerve or other pathways^14,15,47^. These inflammatory factors, pathological α-synuclein, and microbial metabolites lead to the dysfunction and even death of dopaminergic neurons, eventually causing PD.

In conclusion, we presented the largest-to-date meta-analysis of the microbial community in the gut of PD, including 16S rRNA gene and shotgun metagenomic data simultaneously. The results showed that potential pro-inflammatory bacteria and genes in PD were significantly increased, while potential anti-inflammatory bacteria and genes were significantly reduced. These changes may result in decreased levels of SFCAs, which may have anti-inflammatory effects, and increased levels of lipopolysaccharides, H_2_S and glutamate, which may have pro-inflammatory effects. Furthermore, RF models can predict PD with high accuracy based on 11 genera (>80%) or 6 genes (>90%) associated with inflammation. Finally, we proposed a potential mechanism to clarify how inflammation contributes to PD. We believe that inflammation may be a future therapeutic target for PD.

## Methods

### Data collection

After quality control (for details, see below), metadata related to PD from 7 countries was collected from 11 studies with 2269 16S rRNA gene amplicon samples (1373 PD and 896 healthy controls) and 236 shotgun sequencing metagenomic samples (122 PD and 114 healthy controls) by searching the keywords “Parkinson” and “microbes” in the National Center for Biotechnology Information (NCBI) SRA database and Google Scholar (Fig. 1). Raw data in this study were obtained from two open-access databases: The European Nucleotide Archive and NCBI SRA database. Details of metadata are provided in Supplementary Table 1, such as BioProject number, country, database, primers, sequencing platform, etc. For the nine 16S rRNA gene studies, four different primer pairs (515F:806R; 314F:806 R; 520F:907R; and 341F:785R) were identified from the metadata, using the V3-V4, V4, and V4-V5 regions to produce amplicons. However, only four of the nine 16S rRNA gene studies we collected provided some confounding factors. Therefore, this study did not control for confounders in our subsequent analysis.

### 16S rRNA gene data processing

According to previous research^52,53^, adapter, barcodes, and low-quality reads (quality score below 20) were screened using Cutadpt v3.4^54^ and paired-end reads were joined using VSEARCH v2.7^55^. To avoid the interference caused by different sequencing regions, the V4 region of all 16S rRNA gene data was extracted using Cutadpt v3.4 with the primer set 520F-785R. Reads <150 bp or samples with fewer than 10,000 reads were removed before OTU clustering. After quality control, all reads were mapped to Greengenes database 13.8 with 97% identity using VSEARCH v2.7 to create the OTU table and assign taxonomy to reference sequences based on the taxonomic information in the Greengenes database^52^. The Greengenes database is comprised of full-length sequences which can further reduce the biased result from different 16 S rRNA gene regions. The OTUs that only appeared in less than one-tenth of all samples were deleted to address PCR biases.

### Shotgun data processing

The quality control process of shotgun data was the same as above. Besides, human reads were removed using KneadData software (https://huttenhower.sph.harvard.edu/kneaddata) with the default parameters. Functional profiling was performed with HUMANn3^29^ using clean reads with default settings based on the UniRef90 database. The associations between genes and microorganisms were obtained from the result of HUMANn3.

MEGAHIT v1.2.9 was used to assemble the clean reads into contigs with the parameters (--min-contig-len 500, --presets meta-sensitive)^56^. Contigs larger than 1500 bp were automated binned by MetaWRAP v1.3.2 (Binning module) with the parameters (--metabat2 --maxbin2 --concoct) to MAGs^57^. dRep v1.4.3^58^ was used to evaluate the completeness and contamination of MAGs. MAGs were first dereplicated using dRep v1.4.3 with the parameters (-comp 80 -con 5), and only high-quality MAGs (completeness > 80% and contamination < 5%) were selected for further analysis. Open reading frames were predicted from MAGs using Prodigal v2.6.3 with default parameters^59^, and genes were assigned to the UniRef90 database for functional annotation using Diamond v2.0.6^60^ with an e-value cutoff of 10^−5^. The taxonomic classifications of MAGs were inferred using GTDB-Tk v1.5^61^ with default parameters. The Maximum-likelihood phylogenetic tree was generated using the IQ-TREE v1.6.12^62^ based on the 120 bacterial (122 archaeal) concatenated ribosomal proteins extracted by GTDB-Tk v1.5 and visualized using Evolview3^63^. Bootstrap values were calculated based on 1000 replicates.

### Statistical analysis

Group differences in taxonomy and gene profile were analyzed using the Wilcoxon rank-sum test. In this study, the results of all multiple comparisons with *p* < 0.05 were considered statistically significant, using the Benjamini-Hochberg (BH) method for *p*-value correction. The correlations among OTUs were calculated using R based on Spearman’s rank correlation (*r* > 0.7 and *p* < 0.05). The OTUs that only appeared in less than one-tenth of all samples were removed before the calculation of correlations. Co-occurrence networks were established using Gephi v0.9.2^64^. PCoA was performed using the R package “vegan” based on the Bray-Curtis distance and analysis of similarities (ANOSIM) was used to determine whether the difference is significant after PCoA.

### Model building based on machine-learning

To better distinguish PD patients from healthy controls, three well-established machine-learning algorithms (i.e., LR, SVM, and RF^52^) were performed using tenfold cross-validation by R to construct models using the abundances of genus or genes. In the process of model construction, the combined amplicon (A1–9) or metagenomic (M1–2) data were first divided into ten parts. Then nine parts (training set) were randomly selected and used for model construction, and then the remaining independent part (test set) was used for model validation. After that, the ROC curve and AUC were used to evaluate the model performance. The importance of each feature in the model was assessed by the R package “randomForest”. Then, the features were sorted by importance, and different numbers of features were selected for modeling to determine the most concise model. The detailed code and documentation for model building are available on GitHub (https://github.com/Yuange-lab/Shiqing-Nie).

### Reporting summary

Further information on research design is available in the Nature Research Reporting Summary linked to this article.

## Supplementary information

Supplementary materials

Supplementary tables

Reporting Summary

## Data Availability

654 MAGs have been deposited into the China National GeneBank DataBase (CNGBdb) with accession number CNP0002780.

## Code availability

The detailed code and documentation for model building are available on GitHub (https://github.com/Yuange-lab/Shiqing-Nie).
