# Supplementary material for: Inflammatory microbes and genes as potential biomarkers of Parkinson’s disease
Source: NPJ Biofilms Microbiomes. 2022 Dec 24;8:101. doi: 10.1038/s41522-022-00367-z (PMC9789082; doi:10.1038/s41522-022-00367-z)
Supplement: Supplementary file 1 — Supplementary materials [file 41522_2022_367_MOESM1_ESM.pdf]

Supplementary files for

Inflammatory microbes and genes as potential biomarkers of Parkinson's disease

Shiqing Nie[1,2], Jichen Wang[1,2], Ye Deng[2,3], Zheng Ye[4], Yuan Ge[1,2,*]

[1]State Key Laboratory of Urban and Regional Ecology, Research Center for Eco-Environmental Sciences, Chinese Academy of Sciences, Beijing 100085, China

[2]University of Chinese Academy of Sciences, 100049 Beijing, China

[3]Key Laboratory of Environmental Biotechnology, Research Center for Eco-Environmental Science, Chinese Academy of Sciences, Beijing 100085, China

[4]Institute of Neuroscience, Center for Excellence in Brain Science and Intelligence Technology, Chinese Academy of Sciences, Shanghai 200031, China

[*]To whom correspondence may be addressed. Email: yuange@rcees.ac.cn

Competing interests

The authors declare no competing interests.

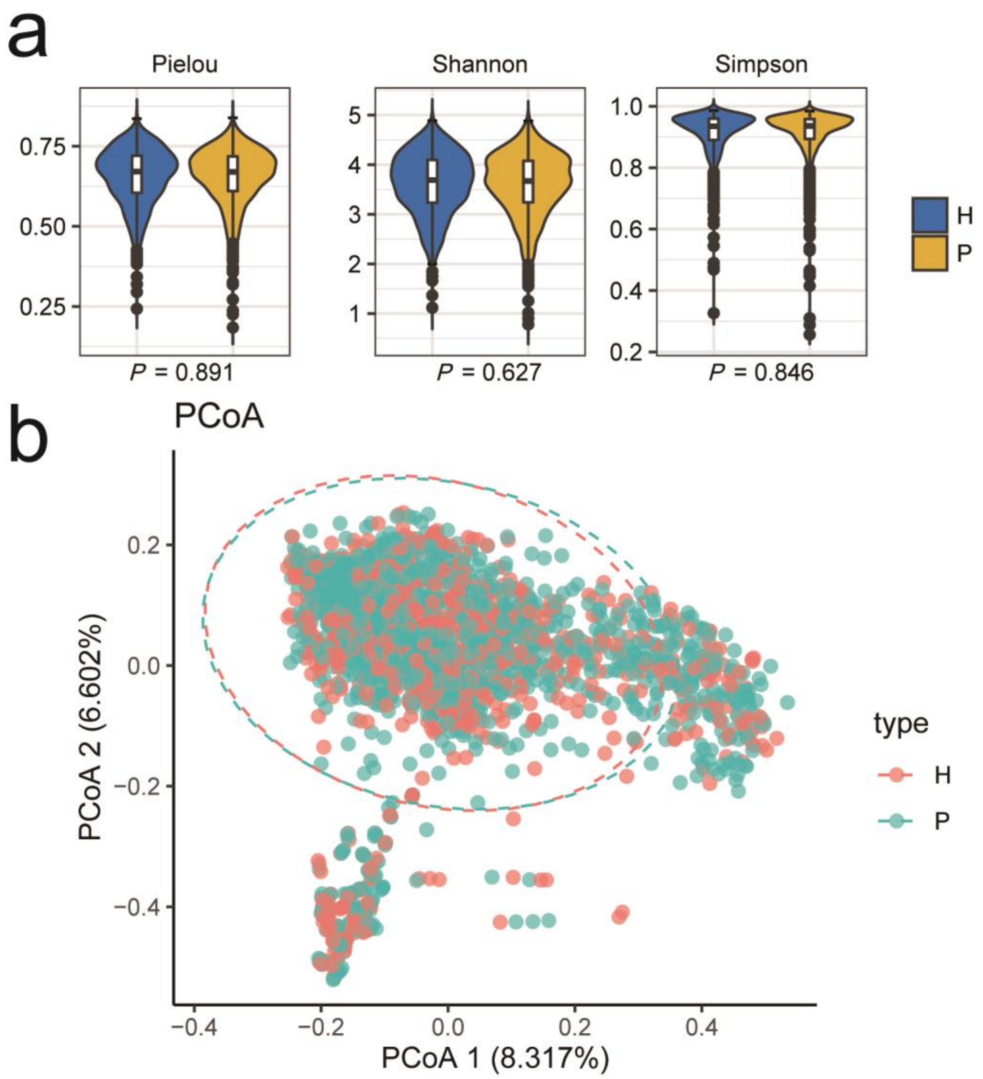

Supplementary Figure 1: The comparison of microbial diversity between Parkinson's disease patients and healthy controls. (a) Alpha diversity indexes. (b) Principal coordinates analysis (PCoA) based on Bray-Curtis distance. H (Healthy control). P (Parkinson's disease patient).

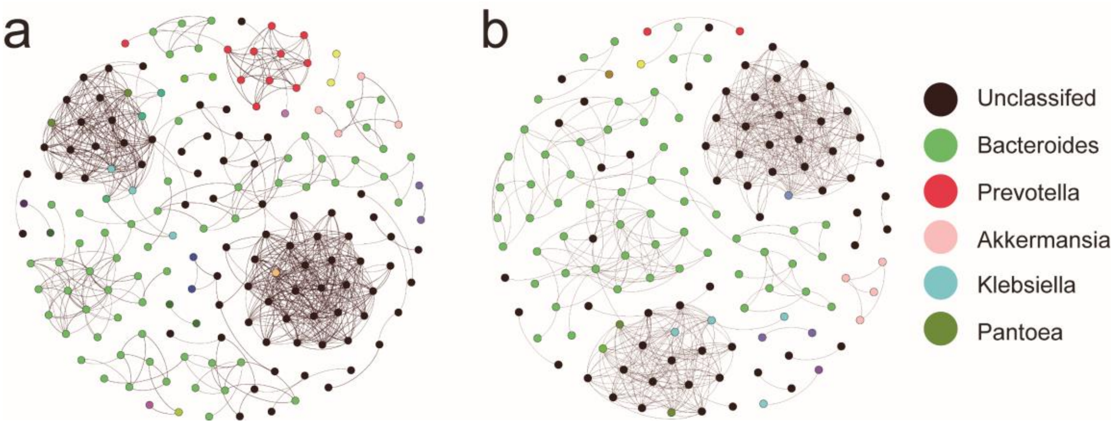

Supplementary Figure 2: Co-occurrence networks. Co-occurrence networks were calculated based on Spearman correlations at the operational taxonomic unit (OTU) level. Only strong ($r >$ 0.7) and significant ($p < 0.05$) correlations are shown in the figure. (a) Healthy controls. (b) Patients with Parkinson's disease (PD). The difference between this figure and Fig. 5 is that the nodes with few connections (< 5) were not removed and the nodes in this figure were colored by genus. On the right of the graph, only the unclassified and five genera with the highest number of nodes were labeled.

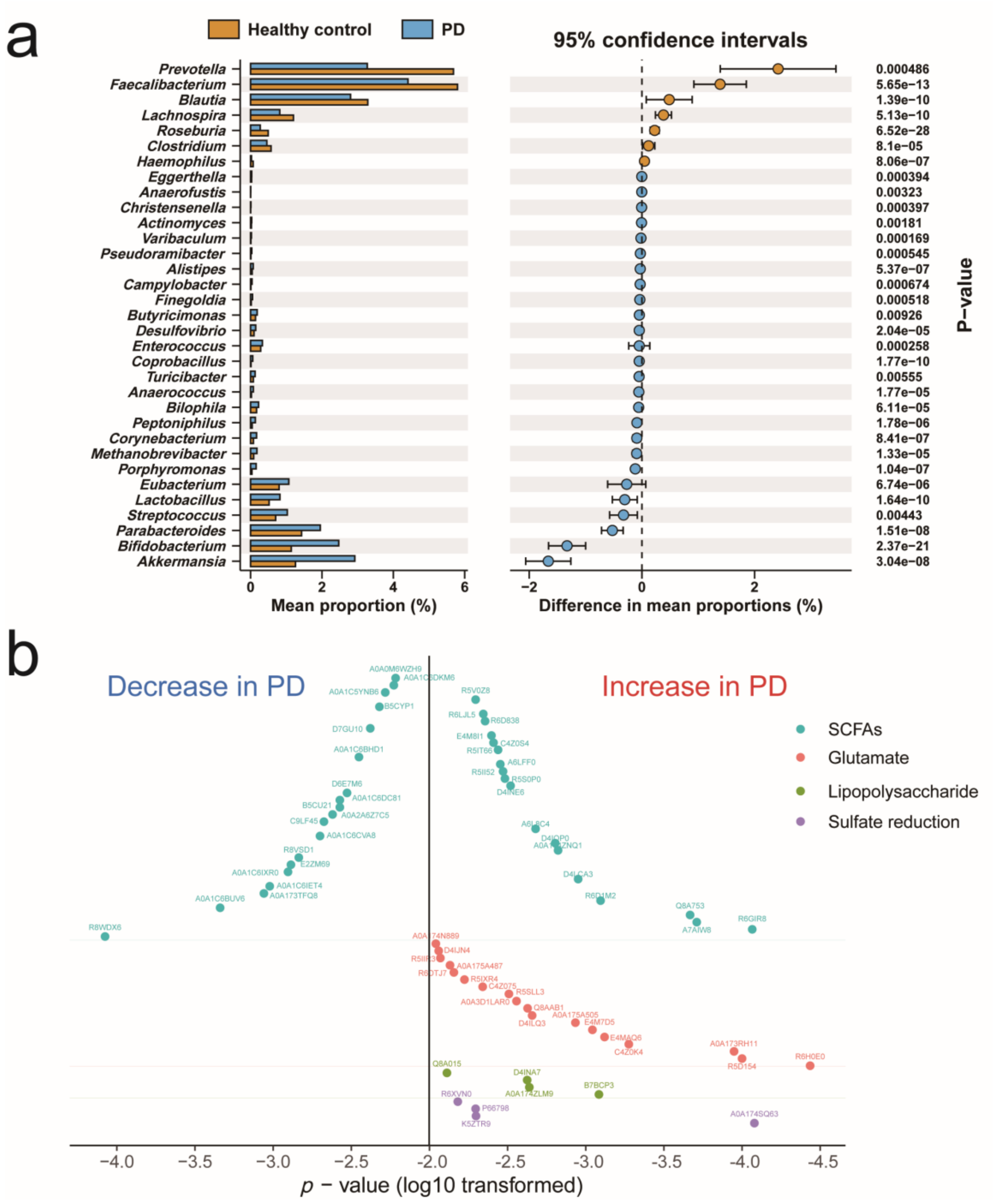

Supplementary Figure 3: Differences in microorganisms and genes between healthy controls and patients with Parkinson's disease (PD). (a) Genera with significant differences in relative abundance. (b) Genes (related to inflammation) with significant differences in transcripts per million (TPM) abundance. SCFAs: short-chain fatty acids.

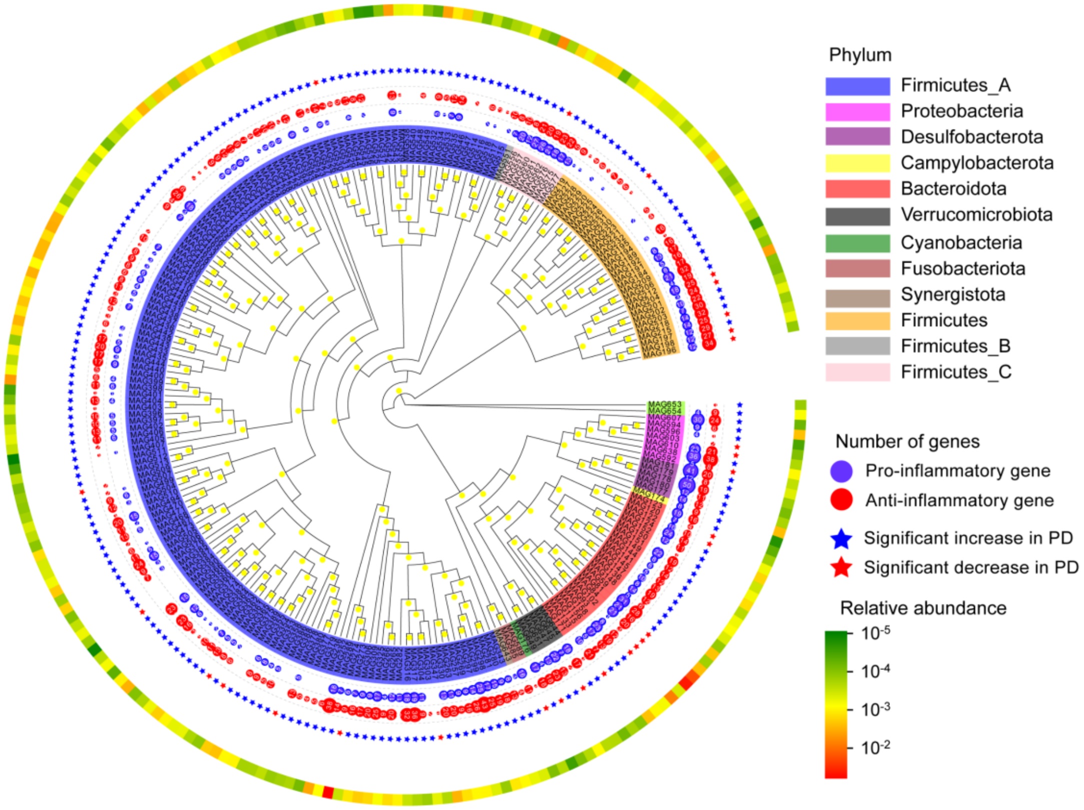

Supplementary Figure 4: The maximum-likelihood phylogenetic tree. The tree was based on the 120 bacterial concatenated ribosomal proteins, colored by phyla. Bootstrap values were calculated based on 1000 replicates, and the value higher than 80% were marked in yellow. The size of the blue (pro-inflammatory gene) and red (anti-inflammatory gene) circle indicates the number of inflammation-related genes. Blue (significant increase in PD) and red (significant decrease in PD) stars indicate significant differences in the relative abundance of metagenome-assembled genomes (MAGs). Parkinson's disease (PD).

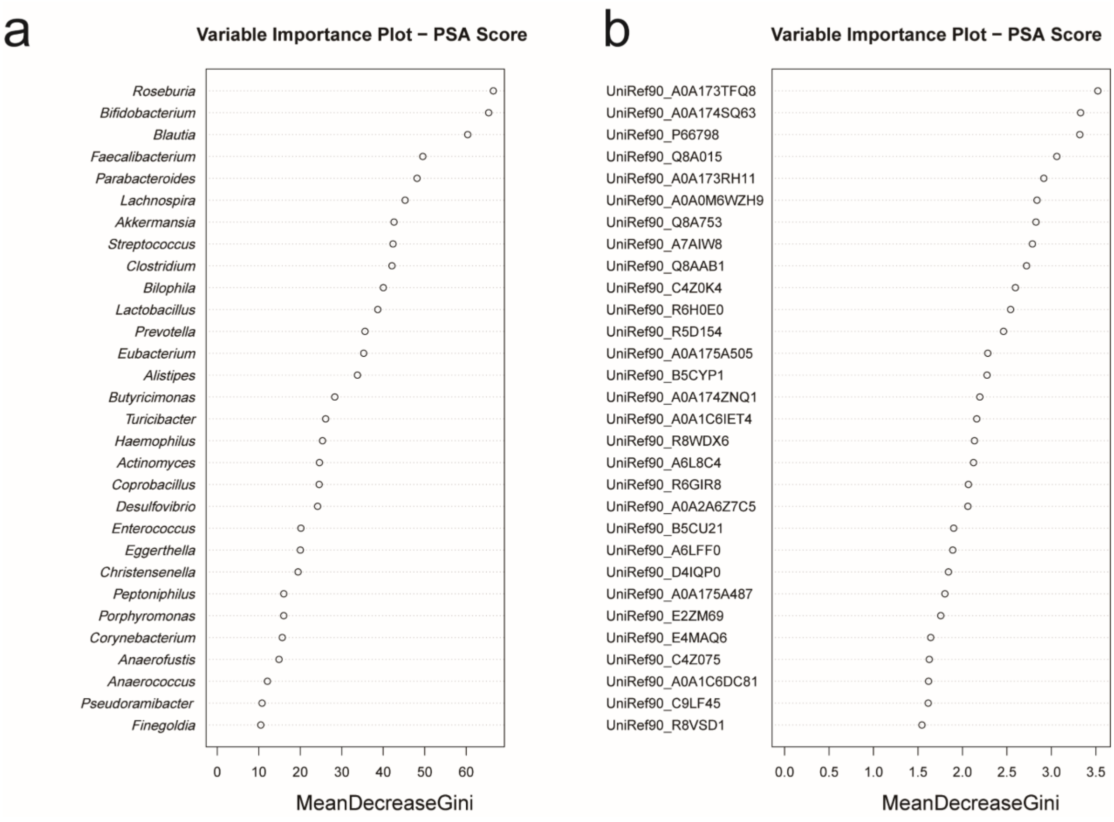

Supplementary Figure 5: The importance of variables in the random forests model. (a) 32 genera. (b) 63 genes. The MeanDecreaseGini values were calculated by the R package "randomForest".

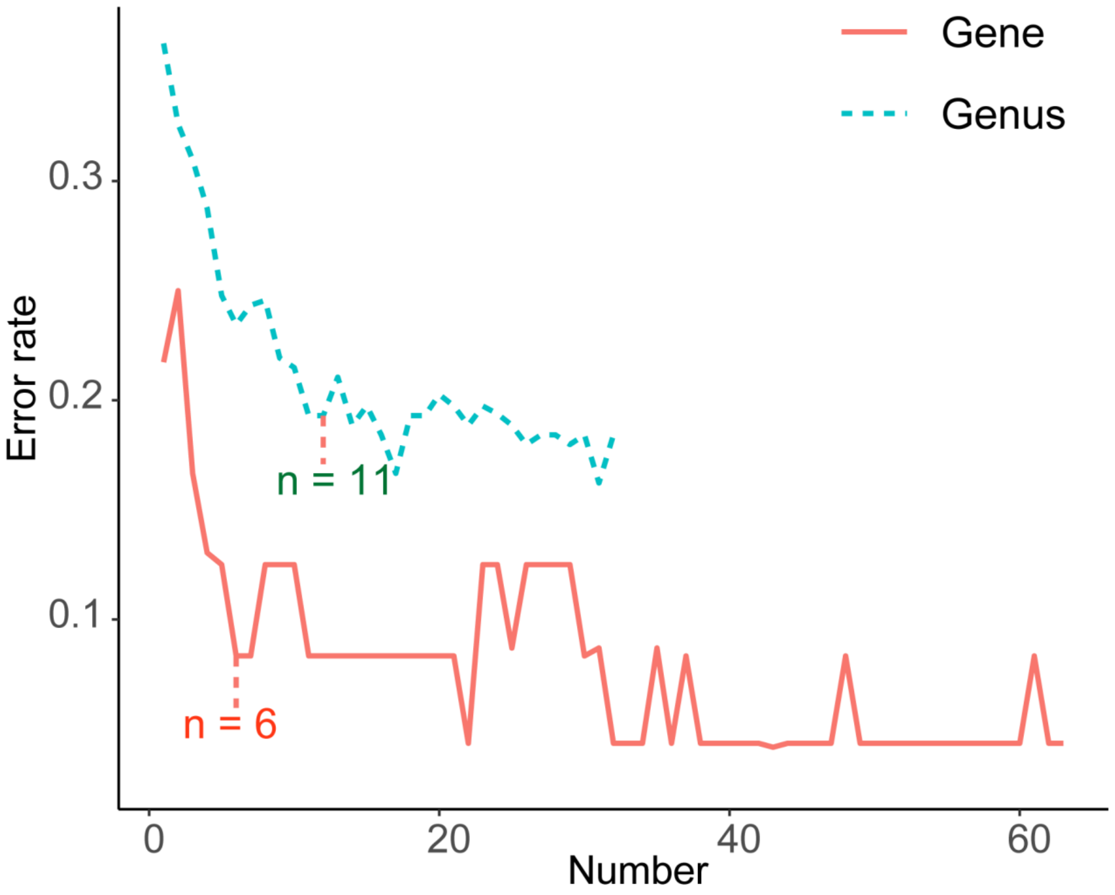

Supplementary Figure 6: Determination of the optimal number of variables in the random forests model. The optimal random forest models can be obtained when selecting the 11 genera or the 6 genes.

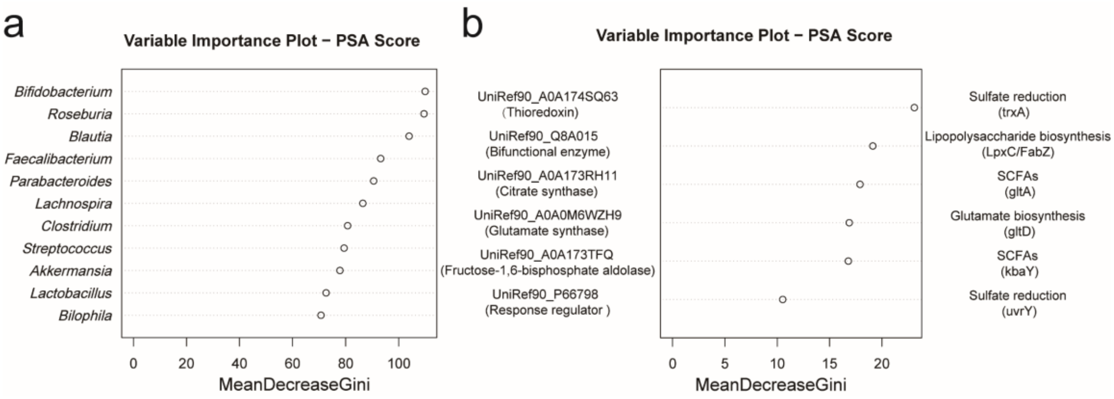

Supplementary Figure 7: The importance of variables in the random forests model. (a) 11 genera. (b) 6 genes. The MeanDecreaseGini values were calculated by the R package "randomForest". SCFAs: short-chain fatty acids.
